# Artificial intelligence for dental implant classification and peri-implant disease detection: A clinical study

**DOI:** 10.6026/973206300212361

**Published:** 2025-08-31

**Authors:** Gousia Shafat Khan, Syed Aaliyah, Sunil Pal, Rahul Tiwari, Smitha M., Heena Dixit, Prashant M.C, Thoid Ali

**Affiliations:** 1 Department of Prosthodontics, Crown & Bridge, Government Dental College and Hospital Srinagar, Srinagar, Jammu and Kashmir, India; 2 Department of Prosthodontics, ITS Dental College, Ghaziabad, Uttar Pradesh, India; 3 Department of Oral and Maxillofacial Surgery, RKDF Dental College and Research Centre, Sarvepalli Radhakrishnan University, Bhopal, Madhya Pradesh, India; 4 Department of Prosthodontics, NSVK SV Dental College, Bengaluru, Karnataka, India; 5 Commisionerate of Health and Family Welfare, Government of Telangana, Hyderabad, India

**Keywords:** Artificial intelligence, dental implants, peri-implantitis, diagnostic imaging, deep learning

## Abstract

Artificial intelligence (AI) is transforming diagnostic accuracy in dental care, particularly in the field of implantology. A prospective research was conducted on 150 patients with 212 previously placed dental implants. The AI system correctly classified 97.2% of implants, with a sensitivity of 96.3% and specificity of 98.0%. For peri-implant disease detection, the system achieved 91.8% sensitivity and 93.4% specificity, with an overall AUC-ROC of 0.94. AI demonstrates high diagnostic performance in both implant classification and peri-implant disease detection.

## Background:

Artificial intelligence (AI) has emerged as a transformative force in modern dentistry, especially in diagnostic imaging and clinical decision-making. Its application in dental implantology is rapidly advancing, particularly for implant classification and the detection of peri-implant diseases. These diseases, including peri-implant mucositis and peri-implantitis, pose significant challenges due to their often asymptomatic progression and reliance on radiographic and clinical findings for early diagnosis. Traditional diagnostic methods, while widely practiced, are often limited by human variability and diagnostic subjectivity, which can delay timely interventions [1, 2-3]. Recent advancements in AI-specifically deep learning and CNNs-have shown promise in analyzing radiographic data to automatically detect the presence, position and condition of dental implants with high precision. Moreover, these models can be trained to differentiate between healthy peri-implant bone and pathological changes, thereby supporting clinicians in detecting early signs of bone loss or inflammation [4, 5-6]. Integrating AI into clinical workflows could substantially reduce diagnostic errors, improve patient monitoring, and standardize classification protocols across varying levels of clinical expertise. The demand for implant treatments continues to rise globally, increasing the need for efficient systems that can handle large volumes of diagnostic images with consistency. AI-based diagnostic tools offer scalable solutions that can process panoramic radiographs or CBCT images rapidly, assisting practitioners in real-time assessments of implant success or failure risk [7, 8- 9].

Despite the potential, few clinical studies have evaluated the real-world performance of AI tools in identifying implant types and diagnosing peri-implant pathology. The findings could contribute to establishing AI as a reliable adjunct in implant dentistry [10]. Therefore, it is of interest to bridge that gap by clinically validating an AI-based system for its diagnostic accuracy in implant classification and peri-implant disease detection in a diverse patient cohort.

## Materials and Methods:

This prospective clinical research was conducted at a tertiary dental care center over a period of 12 months, following institutional ethical clearance and patient consent. A total of 150 patients with previously placed dental implants were recruited. Inclusion criteria comprised patients aged 20-70 years with at least one endosseous dental implant placed more than 6 months prior. Exclusion criteria included patients with systemic conditions affecting bone metabolism, recent antibiotic use, or incomplete radiographic records.

Each patient underwent a standardized intraoral examination and digital imaging with CBCT. The acquired DICOM images were processed through a pre-trained CNN -based AI model. This model was designed to classify implants (e.g., tapered, cylindrical, or blade-form) and detect peri-implant bone defects indicative of mucositis or peri-implantitis. The AI model output was compared with ground truth diagnoses established by two experienced oral radiologists blinded to the AI results. Accuracy, sensitivity, specificity, and AUC-ROC were computed for both implant classification and disease detection. Data were analyzed using SPSS version 26.0. Chi-square tests and ROC curve analyses were applied. A p-value of <0.05 was considered statistically significant.

## Results:

Out of 150 participants, 90 were male and 60 were female, with a mean age of 48.6 ± 10.2 years. A total of 212 dental implants were evaluated. The AI model successfully classified 206 implants correctly, showing an overall classification accuracy of 97.2%. Sensitivity and specificity for identifying implant types were 96.3% and 98.0%, respectively. The AI system showed particularly high performance in detecting cylindrical implants, followed by tapered types. Misclassifications occurred in cases with image artifacts or overlapping anatomical structures (Table 1 - see PDF). For peri-implant disease detection, the AI system demonstrated an overall sensitivity of 91.8% and specificity of 93.4% when compared to clinical and radiographic findings validated by two independent oral radiologists. It correctly identified peri-implant mucositis in 48 of 52 cases and peri-implantitis in 28 of 30 cases. The AUC-ROC for overall disease detection was 0.94, indicating excellent diagnostic capability (Table 2 - see PDF).

## Discussion:

The present research highlights the clinical utility of AI in dental implantology, specifically for implant classification and peri-implant disease detection. The AI system demonstrated a high degree of diagnostic accuracy, with over 97% correctness in implant classification and excellent sensitivity and specificity in identifying peri-implant pathology. These findings align with prior evidence indicating the robustness of CNNs in processing dental radiographic images with minimal human oversight [11]. One of the most significant advantages of AI in this context is its ability to standardize diagnostic protocols, minimizing inter- and intra-observer variability. In traditional settings, diagnostic outcomes often differ based on the clinician's experience and interpretation. The use of deep learning algorithms, however, ensures consistency across various clinical cases by learning from a vast dataset of annotated images and mimicking expert-level reasoning [12]. In the current research, the most accurate results were seen in cylindrical and tapered implants, likely due to the abundance of such types in training datasets, reflecting real-world clinical distribution. Moreover, the AI's performance in detecting peri-implant mucositis and peri-implantitis was highly reliable. Early detection of peri-implant inflammation is essential to prevent irreversible bone loss, and timely interventions can significantly improve implant prognosis. Traditional diagnostic methods rely heavily on probing depth and radiographs, which may not always reflect subtle changes in bone or soft tissue. AI, by contrast, can enhance detection by identifying nuanced radiographic patterns that may be overlooked by the human eye [13]. Interestingly, false positives in disease detection were more frequent in images with metal artifacts or anatomical overlaps, a known limitation of both conventional radiographic interpretation and AI processing. Nevertheless, the model's high AUC-ROC of 0.94 indicates substantial clinical potential. The model also functioned well in diverse patient demographics, suggesting generalizability across age groups and implant types [14, 15]. While this research supports AI as an adjunct tool, it does not propose replacing clinical expertise. Instead, it reinforces the idea of AI serving as a diagnostic co-pilot, improving workflow efficiency and diagnostic confidence. Future improvements should focus on refining algorithms to address limitations related to image quality and incorporating clinical variables such as plaque scores or bleeding indices for a more holistic diagnosis [16, 17, 18, 19- 20].

## Conclusion:

The application of AI in dental implant classification and peri-implant disease detection with high diagnostic accuracy is validated. Incorporating AI into routine dental practice could significantly improve patient outcomes, particularly in the early management of implant-related complications. Continued algorithm development and multi-center validations are essential for widespread clinical adoption.
